# M-AAA-nsplaining: Gender bias in questions asked at the American Anthropological Association’s Annual Meetings

**DOI:** 10.1371/journal.pone.0207691

**Published:** 2019-01-18

**Authors:** Jeffrey Winking, Allison L. Hopkins, Michelle Yeoman, Cory Arcak

**Affiliations:** 1 Department of Anthropology, Texas A&M University, College Station, Texas, United States of America; 2 Department of Veterinary Integrative Biosciences, Texas A&M University, College Station, Texas, United States of America; 3 MSC L.T. Jordan Institute for International Awareness, Texas A&M University, College Station, Texas, Unietd States of America; Stellenbosch University Faculty of Medicine and Health Sciences, SOUTH AFRICA

## Abstract

A large body of research has revealed the challenges that disproportionately affect women as they climb the academic ladder. One area that has received relatively little attention is women’s experiences at academic conferences, which are often integral to academics’ professional development. As conferences are attended by professional colleagues and influential players in specific fields, the professional consequences of any gender bias in criticism are likely to be amplified at such venues. Here, we explore the degree to which the likelihood of audience members asking a question and offering criticism is associated with the gender of a presenter. Audience questions were tabulated during the authors’ visits to the three American Anthropological Association Annual Meetings. The results suggested that men were indeed marginally more likely to ask a question, both when considering all types of questions and when considering only critical questions. However, there was no evidence that they differentially targeted women for these questions. Future research might explore what motivates assertive and critical speech in men and women and how their experiences in receiving it might differ, particularly in academic settings in which critical speech might be considered more acceptable.

## Introduction

Women continue to be underrepresented in numerous academic fields, and importantly, remain underrepresented in higher academic ranks [1–5]. A large body of research has focused on the various causal factors that perpetuate these inequalities. Many of these involve social and institutional impediments, such as gendered expectations of labor distribution in the office and the home (e.g, [6, 7]), but they also include individual biases that lead women and men to be treated differently in their professional interactions with others (e.g., [8]).

One area where women likely face hurdles is at academic conferences [9]. Conference attendance is often integral to academics’ professional development—they present unique opportunities to receive feedback on recent research, learn of others’ groundbreaking work, and forge collaborations.

As one of the few venues in which academics have the opportunity for face-to-face conversation, women might experience another form of gender bias which they are free from in academic writing—bias that relates to gender differences in patterns of speech [10–12]. One purported form of such biased behavior is popularly known as “mansplaining,” in which a man asserts his perceived superior understanding of a topic when addressing a woman, even though she has established expertise in that topic [13, 14]. However, no study has formally assessed whether such a pattern results in women receiving disproportionate criticism, particularly by men. Here, we explore whether this occurs at the American Anthropological Association Annual Meeting. The conference venue is an important location for capturing gender bias in criticism for two reasons: 1) it is a public forum attended by colleagues and thus any effects of gender bias in criticism would be amplified, and 2) the question/answer portion of conference presentations provides an ideal opportunity to tabulate discrete critiques, unlike those offered during natural conversation.

Gender and Speech

The study of gendered patterns of speech has a long and complex history. Early attempts were founded upon little more than anecdote and speculation, much of which was rooted in sexist stereotypes of the time (e.g., [15–17]). The late twentieth century saw the development of a research industry focusing on tabulations of observed speech behaviors and the examination of how these differed across gender and other social factors (e.g., see citations within [11, 12]). There was a great deal of variation in the reported effects, with some reporting significant effects that differed not only in magnitude but in direction compared to other studies (e.g., for tag questions such as " …, isn't it?", see [18, 19]).

Much of the variance in the literature was due two major challenges that muddled research design—those relating to function and to context [20]. Regarding function, it has proven challenging to define meaningful categories of speech behaviors that share a common function. For instance, is an interruption a ploy to seize the floor or a cooperative overlap that promotes cohesion between speaker and listener [11]? Additionally, it is difficult to isolate all of the relational and environmental factors that co-vary with gender, and which might be the true drivers of any observed variance. If social status, for example, affects speech patterns *and* is associated with gender, a spurious gender effect might be reported unless social status is appropriately accounted for [21]. There are likely a number of unknown variables that associate with both gender and speech that could lead to such false positives. Similarly, environmental contexts, such as whether the study takes place in a classroom or a movie theatre, can lead to major differences in speech patterns in men/boys and women/girls. Increasingly, researchers have embraced this complexity, moving away from a simple difference-model and toward more context-rich explorations of language and gender [22, 23]. This shift of course does not negate the importance of empirical studies in illuminating gender biases, but highlights the need to take contextual factors into account and to be wary of over-generalization.

Another challenge of the tabulation approach was simply the results: meta-analyses run over large numbers of empirical studies ultimately revealed mostly negligible effects [12, 23, 24]. Some effects do seem to hold, however. For instance, male speakers tend to engage in more assertive speech, while women tend to exhibit more affiliative speech and related behaviors (e.g., smiling) [12, 24]. While the calculated effects are quite small, the degree to which the two categories of speech represent poles of a single dimension would amplify the effect. Similarly, men tend to spend more time talking in mixed groups [10, 12, 25], and engage in intrusive interruptions more frequently [11]. These effects are often interpreted as supporting the “dominance” viewpoint of language differences, which suggests that these differences result from (and perpetuate) a patriarchal social order.

A phenomenon that is widely discussed in the media today, and which is rooted squarely in this dominance viewpoint, is that of “mansplaining” [13]. This is the tendency for men to offer critical, explanatory, or informing comments to women, even if the female listener has established expertise on the topic being discussed. Despite this being a widely-discussed gender bias, few systematic studies have reviewed this phenomenon. We attempt to fill this void here through an exploration of questions asked at a professional academic conference (AAA Annual Meetings).

### Gender in Anthropology, the AAA, and its Annual Meetings

The AAA has 9,218 members, and while demographic data is not collected in the application process, a recent survey of members suggests that women constitute a majority of the membership at 61% (the remaining include 36% men and 2% not selecting a binary option) [26]. This figure is comparable to the percentage of Anthropology doctorate degrees earned by women from 2002 to 2012 (60%), a figure that is marginally higher than social sciences in general (57%) and one that grew during this period [27]. Of those who participated in the AAA membership survey, women represented a majority in all academic ranks, although there was a decline in women’s representation as academic rank increased (Table 1) (Ginsberg, 2016 AAA Member Survey unpublished data). However, a tabulation of faculty members of the 79 Ph.D.-granting programs that were ranked by the National Research Council in 2010 revealed that women constituted the majority of assistant (58.9%, n = 319) and associate (50.9%, n = 544) professors, but not of full professors (39.1%, n = 778) (Table 1).

**Table 1:** Gender and academic rank for 2016 AAA survey and meeting presenters.

	All	Student	Non-TT Academic	Asst. Prof	Assoc. Prof	Full Prof
AAA 2016 Survey						
Female	1184 (61)	295^a^ (70)^b^	127 (67)	139 (70)	140 (68)	133 (52)
Male	701 (36)	126 (30)	62 (33)	58 (30)	66 (32)	123 (48)
Other/Prefer not to answer	43 (2)					
TT Profs in Ph.D. Programs						
Female	769 (47)			188 (59)	277 (51)	304 (39)
Male	872 (53)			131 (41)	267 (49)	474 (61)

^a^The totals do not sum to figures in “All” column because only those whose status is known and who are active in academia are included in academic rank totals).

^b^Percentage of relevant gender within relevant category.

While women in anthropology have greatly increased their representation through time, they still appear to disproportionately face challenges in their professional development. Female anthropology professors reported less satisfaction with university support, mentor support, and departmental collegiality than their male colleagues. They also report greater challenges due to gendered expectations of labor distribution, both with more administrative duties in the office and family responsibilities in the home [7].

Similarly, women and men likely experience academic conferences differently. For professional anthropologists, the AAA Annual Meeting is the largest annual conference with attendance that tends to range in the upper thousands [28]. Research suggests that women in academia are less frequently invited to be members of symposia [9], and only recently have scientific organizations begun to take seriously the threat of sexual harassment at these venues [29]. Furthermore, women appear to exhibit more of an orientation towards less prestigious presenting options [9, 30] and are more likely to decline speaking invitations [31]. The reasons why female academics exhibit such behavioral patterns are likely complex and deeply embedded in the cultural norms surrounding gendered expectations of assertiveness as well as biased distributions of professional and family responsibilities [31]. Overall, the conference experience might be less positive for women due to these numerous factors ([29], although see [32]). Additionally, if there does exist a pattern of increased criticism directed at women, this might further discourage participation.

### Conference questions and hypotheses

The most common format for sessions at the AAA Annual Meeting is for each speaker to present during a 15 minute time slot and for all questions to be held until all speakers have presented, at which time the questions are asked publically. Such a context offers a unique opportunity for exploring speech content, as it is a natural setting in which speech is presented in discrete segments and directed to specific receivers. Furthermore, any bias is likely to be amplified in effect, as the critical/explanatory speech is witnessed by many individuals and by those who often carry disproportionate influence in particular fields. However, like in much of the literature, these types of interactions are complicated by relational and motivational ambiguities. Although speaking on a topic establishes one as a de facto authority, audiences are also often comprised of like-interested specialists, and the true status relationship might be lost on observers. Similarly, an academic conference is one of the few venues in which critical speech might actually be sought or encouraged as researchers hone methodological approaches and negotiate inferences, making the motivation for critical speech ambiguous.

Regardless of these complications, a number of hypotheses (H) and associated predictions (P) can be derived:

(H1) If men speak more in mixed groups than women [12], then

(P1) men should be more likely than women to ask questions in general in mixed audiences.

(H2) If men are more likely to assume authority on a given topic and speak accordingly in interactions with women [13], we can begin with a liberal inference of authority by assuming that if a person asks any question or makes any comment in such a professional setting, they are assuming authority. Thus, we can predict that

(P2a) men should be more likely to offer any question/comment to a female presenter than to a male presenter, and

(P2b) men should be more likely to offer any question/comment to a female presenter than a female audience member would.

Using a more strict definition of assuming authority, we can also predict that

(P3a) men’s questions/comments to female presenters will be more likely to be critical than those to male presenters, and

(P3b) men’s questions/comments to female presenters will be more likely to be critical than those from female audience members.

These effects combined should result in

(P4a) men being more likely to offer a critical question/comment to female presenters than to male presenters, and

(P4b) men being more likely to offer these questions/comments than female audience members.

The reciprocal predictions of H2 (women will be more likely to assume authority when interacting with male presenters) will be explored to assess whether any gender bias flows in the opposite direction as well.

## Methods

### Data collection

Data was collected during the American Anthropological Association Annual Meetings (AAAs) in 2014 (Washington, D.C.), 2015 (Denver, Colorado), and 2016 (Minneapolis, Minnesota). Convenience samples of sessions were attended. Sessions with relatively even numbers of male and female presenters and with discussions scheduled as the final block were sought, although other factors, such as proximity and even coder interest influenced selection. At the AAA conference, each session is typically sponsored by one of 40 different AAA sections, which are organized around specific topics (e.g., Society for Medical Anthropology). The sessions that were sampled include 20 different section sponsors, with the most sampled section including six sessions. While the sample is clearly not a probability sample, we were not attempting to characterize the nature of the conference as a whole, but instead examine within-session treatment effects. Thus we do not believe this hampered the study’s inferential value.

If questions were solicited after a specific presentation, only data from that presentation was included. In most sessions, however, questions were reserved until the final discussion block. No details of participants other than gender were recorded, and speech was only categorized and not recorded. Human subjects review and approval, including a waiver of consent, was granted by the Texas A&M University Institutional Review Board under protocol IRB2015-0662. Permission was also granted by AAA Annual Meeting coordinators.

Coders positioned themselves toward the back of the room so as to be able to view and hear as best as possible, but still remain inconspicuous. Prior to the question round, the number of men and women in the audience was tabulated. Although gender was not difficult to ascertain in the vast majority of cases, if a dichotomous gender category was not being performed or immediately evident, individuals were assigned an “other / unknown” category. For each round, the number of hands raised by men and women was recorded, as well as the gender of the person selected, the speaker targeted for their comment, and the nature of the question. Occasionally questions were directed to the group of presenters, instead of one individual, which was also recorded. Individuals were monitored to determine if the same audience member asked more than one question in order to record the data accordingly.

Questions were categorized into any combination of seven categories (Criticism, Alternative Interpretation, Reference, Request to Expound, Request to Clarify, Compliment, Other). This process was repeated until the question/answer portion ended. For this study, a question/comment was deemed to be “critical” if it included a Criticism (a declarative criticism of the methods, interpretation, etc., e.g., “I don’t think such a forum would elicit natural behavior…”), Alternative Interpretation (an offering of a different way of conducting the study or interpreting the results, e.g.,“The effects might also reflect that…”), or Reference (pointing the presenter to a relevant reference, e.g., “Are you familiar with the paper that just came out by….”). In contrast, questions were deemed “non-critical” if they were only categorized as one or more of the following: Request to Expound (any suggestion or question relating to further information that does not directly relate to the conclusions of the research, e.g., “Why do you think they were acting like that?”), Request to Clarify (any question to clarify something that was stated, e.g., “How did you decide which sessions to attend?”), Compliment (a compliment of the presentation, e.g., “That was a great talk, very interesting…”), and Other (any major comments that do not fit under these categories).

All data coders (JW, AH, and MY) trained using online recordings of academic presentations which included question rounds. Seven presentations with 26 questions were coded by all data coders. This resulted in an average pairwise agreement of 82.1% and a Fleiss’ kappa of 0.764 for Criticism, 92.3% and 0.639 for Alternative Interpretation, and 87.2% and 0.624 for Reference (the three categories of importance for this paper). These represent acceptable levels of agreement in all reported benchmark configurations, but still warrant some caution in interpretation [33–35].

### Analysis

The data were organized in four ways in order to test the four types of comparisons. To test for differences in general frequencies of questions (P1), the data were arranged such that each individual represented a single row. To test for differences in how audience members responded to male versus female presenters (P2a and P4a), the data were arranged so that each row represented an audience member “opportunity.” For example, if an audience member had the opportunity to ask a question to a male presenter, that would constitute a row, and if that audience member also had the opportunity to ask a question to a female presenter (in a mixed-gender discussion panel), then that would constitute a second row for that audience member. Audience members thus had either one or two rows, each with an outcome for whether or not the audience member asked any question (P2a) or a critical question (P4a). The data were analyzed using a mixed logistic regression model using PROC GLIMMIX in SAS 9.2 with Audience ID as a nested random effect within the random effect for Session. Controls for the size of the audience, whether or not the question round was during a panel-wide discussion, and the number of panelists of the gender of analysis, were initially included and then eliminated if not significant.

For tests of whether questions were more likely to be critical based on the gender of the asker and the speaker (P3a and P3b), the data were arranged such that each row represented a single question, and the outcome was a dichotomous variable signifying whether the question was categorized as critical. For P3, only questions to single individuals or single-gender groups were included, as those directed to mixed-gender groups could not be coded as being directed to either a male or female. For P4, however, a question asked to a mixed-gender group of presenters would be included in both the database limited to questions asked to women and the database limited to questions asked to men. The data were analyzed using a mixed logistic regression model with no controls and with Audience ID and Speaker ID as crossed random effects. However, the inclusion of Audience ID resulted in convergence problems, suggesting that it captured very little variance, so it was omitted.

Finally, for tests exploring whether the gender of the audience member was associated with the likelihood of asking any question (P2b) or a critical question (P4b), the data were arranged such that each row represented a single individual. The outcome variable represented whether or not they asked any type of question for P2b or a critical question for P6b. The data were analyzed using a mixed logistic regression model with Session as a random effect. A number of controls were initially included and then eliminated sequentially if not significant. These included the size of the audience, whether or not the question round was during a discussion, and the number of panelists in the session who were of the gender of analysis (e.g., when analyzing the likelihood of asking a female presenter a question, this would be the number of female presenters in that session).

## Results

### Descriptive

A total of 45 sessions were attended in which the audience was invited to ask questions. In 36 of these, the questions were solicited during a panel-wide discussion, and for 9, they were solicited after a single presentation (data from subsequent presentations in these sessions was not used). This led to a dataset that includes 195 speakers (114 women, 81 men) and 848 audience members (502 women, 346 men) from whom 175 questions were asked (81 by men, 94 by women). Only five individuals raised their hands and were not called on (three men and two women).

Including all 45 sessions observed for the current study, the overall audience (59% female) and speaker composition (57% female) did not significantly differ from the membership survey results (both comparisons approached significance at p<0.1). Similarly, the gender composition of speakers in our sample trended male, but not significantly so, compared to a random sample of all speakers at the three conferences (n = 1,023, female = 64.8%, χ^2^ = 2.785, p = 0.095). In order to account for the fact that certain sections were oversampled, we also calculated a weighted proportion of the study sample by calculating proportion female within each section and then weighting them by the total number of members within that section. This did not substantially affect the estimates or the analyses (female proportion changed to 60%), which suggests that the convenience sample of sessions is fairly representative of the gender distribution of the organization.

### Hypotheses

Table 2 presents the results of general models without considering speaker gender. In general, audience members were more likely to ask a question when the audience is smaller in number and when the question round is during a discussion. When given the opportunity to ask a question, regardless of the gender of the speaker, men were marginally more likely than women to ask a question of any type (Table 2A). Men’s questions were in the direction of being more critical than women’s questions (using questions as the unit of analysis), but the effect was not significant (Table 2B). Similarly, when examining only critical questions, men were marginally more likely than women to ask a critical question when including questions to both men and women (Table 2C).

**Table 2:** Results of mixed logistic models testing for general gender effects.

	Estimate	S.E.	Signif.
Likelihood of Asking Any Question^a^			
Intercept	-0.167	0.252	0.510
Gender = Woman	-0.363	0.188	0.054
Audience Size	-0.039	0.009	<0.001
During Discussion = 1	1.104	0.332	0.001
Likelihood a Question is Critical^b^			
Intercept	-0.778	0.287	0.008
Gender = Woman	-0.122	0.382	0.750
Likelihood of Asking Critical Question^c^			
Intercept	-2.264	0.238	<0.001
Gender = Woman	-0.573	0.304	0.060
During Discussion = 1	-2.038	0.762	0.008

^a^Unit of analysis = individual audience member. Session included as a random effect. n = 848.

^b^Unit of analysis = question. Speaker ID included as a random effect. n = 174.

^c^Unit of analysis = individual audience member. Session included as a random effect. n = 848.

Table 3 presents the results of the mixed-effects logistic regression analyses testing the six predictions for men and the six predictions for women. The full models are presented in the Supporting Information (S1 and S2 Tables). No predictions were supported. It should be noted that while the non-significant effects were mostly in the predicted direction for men, many of these effects are almost zero. The only significant effect was in the opposite direction—women were *less* likely than men to ask a male speaker a question. Sensitivity analyses were conducted on standard logistic models (without the random effects) for simplicity. While the variance of the random effects ranged from 0.025 to 2.097, we feel using standard logistic models is appropriate, as excluding the random effects had no impact on the direction or significance status of any predictors (the one significant main effect mentioned above does change to one-tailed significant) (Supporting Information, S3 and S4 Tables). The sensitivity analyses suggest that the tests of P2 would detect a small effect with a power of 0.90 and significance level of 0.05 (OR range of the four tests: 1.79–1.99), tests of P3 would detect a moderate effect (OR range for the four tests: 3.60–4.95), and tests of P4 would detect a small to moderate effect (OR range for the four tests: 2.51–3.51) (Supporting Information, S5 Table).

**Table 3:** Results of mixed logistic models testing predicted effects.

	Male Audience Members				Female Audience Members			
	Direction	Estimate	S.E.	Signif.	Direction	Estimate	S.E.	Signif.
P2a: Ask more ?s to opposite sex than to same sex^a^	Opposite	-0.157	0.248	0.527	Opposite	-0.259	0.216	0.231
P2b: Ask more ?s than opposite sex to opposite sex^b^	Predicted	0.245	0.215	0.254	Opposite	-0.512	0.237	0.031
P3a: More ?s to opposite sex critical than to same sex^c^	Predicted	0.380	0.640	0.564	Opposite	-0.349	0.645	0.594
P3b: More ?s than opposite sex’s ?s critical to opposite sex^d^	Predicted	0.042	0.452	0.926	Predicted	0.173	0.575	0.765
P4a: Ask more critical ?s to opposite sex than to same sex^e^	Predicted	0.036	0.410	0.929	Opposite	-0.498	0.398	0.212
P4b: Ask more critical ?s than opposite sex to opposite sex^f^	Predicted	0.277	0.360	0.442	Opposite	-0.519	0.433	0.231

^a^Unit of analysis = Audience member opportunity. Session and Audience Member ID (nested) included as random effects. For men, n = 594; controls include Audience Size and Number of Female Speakers. For women, n = 900; controls include Audience Size.

^b^Unit of analysis = Audience member. Session included as a random effect. For men, n = 747; controls include Audience Size and Number of Female Speakers. For women, n = 747; controls include Audience Size.

^c^Unit of analysis = Question. Speaker ID included as a random effect. Questions directed to entire panels excluded. For men, n = 62. For women, n = 76.

^d^Unit of analysis = Question. Speaker ID included as a random effect. For men, n = 112. For women, n = 98.

^e^Unit of analysis = Audience member opportunity. Session and Audience Member ID (nested) included as a random effect. For men, n = 594; controls include Number of Female Speakers. For women, n = 900, no controls.

^f^Unit of analysis = Audience member. Session included as a random effect. For men, n = 747, no controls. For women, n = 747, no controls.

**Fig 1:**
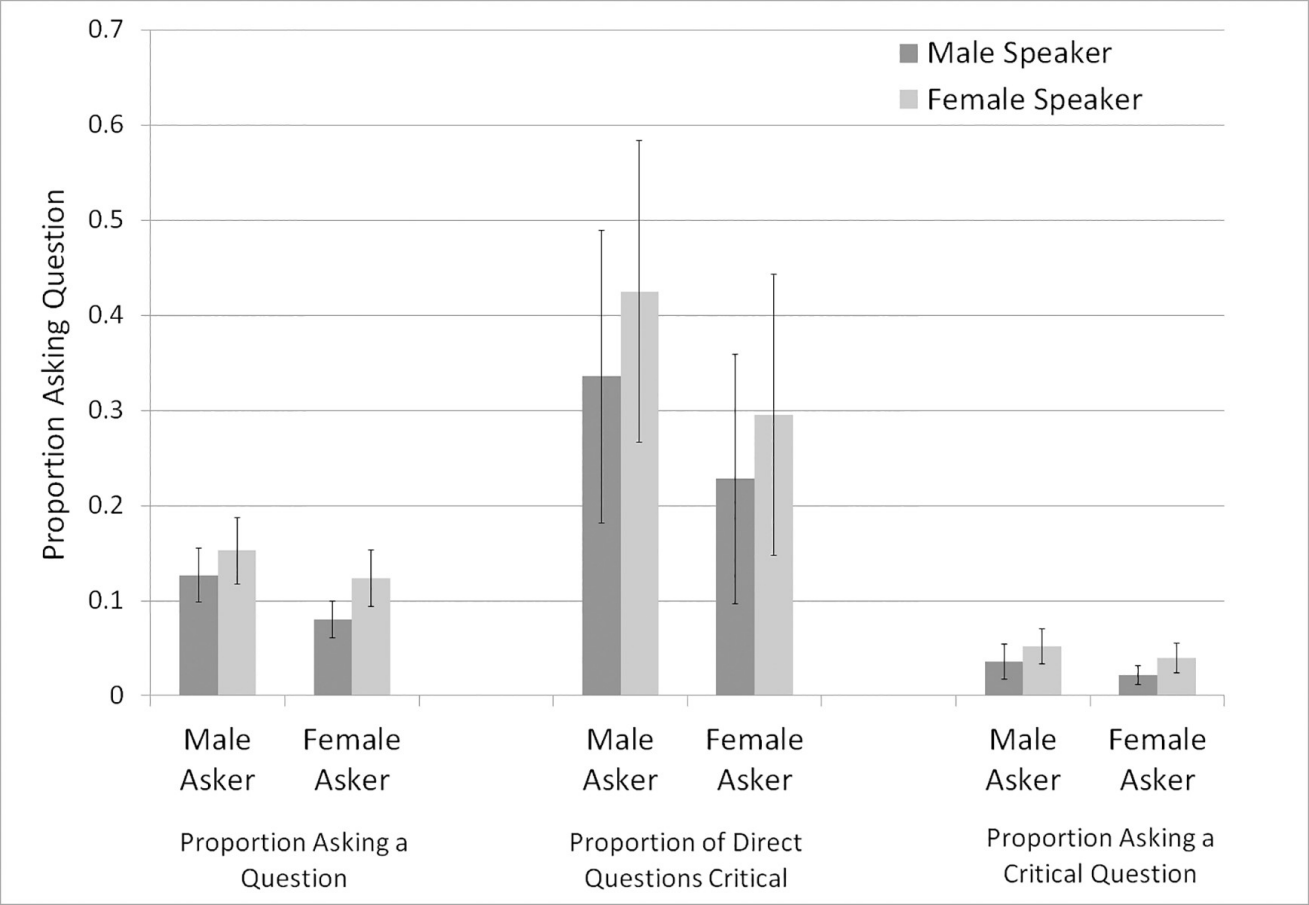
Estimated probabilities of questions being asked by gender of speaker and asker. (Below Fig 1): Estimates are calculated probabilities based on analyses of P2b, P3a, and P4b with controls set to sample means. Error bars are standard errors.

Fig 1 presents the estimated probabilities with significant controls set to sample means. Each probability can be estimated using two analyses. For example, the proportion of men asking women a Methods). The table presents the data as analyzed in P2b, P3a, and P4b, as we believed these to be the question can be calculated from P2a and P2b based on different datasets (see Analysis section in simplest representations of the probabilities. However, one direction flips depending on the dataset used—based on the dataset used to test P2, the probability of men asking a woman a question is estimated to be higher than that for men asking a man a question.

### Contextual factors

In a post-hoc analysis, the gender ratio of the room (audience plus speakers) had no impact on the likelihood of female audience members asking a question. However, male audience members were significantly more likely to ask questions in rooms with *fewer* men (Table 4). Including sex ratio as a control variable in the analyses testing the hypotheses (above), however, did not impact the direction or significance status of any of the main effects. Furthermore, using the speaker as the unit of analysis, the gender ratio of the speaker panel was not associated with the number of questions received by either male or female speakers.

**Table 4:** Results of mixed logistic models testing effect of gender ratio on likelihood of asking question.

	Male Audience Members^a^			Female Audience Members^b^		
	Estimate	S.E.	Signif.	Estimate	S.E.	Signif.
Intercept	-1.629	0.695	0.024	-1.064	0.827	0.205
Percent Female in Room	2.588	1.155	0.026	0.885	1.200	0.461
Audience Size	-0.039	0.012	0.002	-0.042	0.012	0.001
During Discussion	1.307	0.462	0.005	0.677	0.442	0.126

^a^Session included as a random effect. n = 346.

^b^Session included as a random effect. n = 502.

## Discussion

In this study, we found that men were marginally more likely to ask questions/make comments in a conference setting; this was true when considering all questions or just critical questions. This is in line with results from similar studies exploring gender differences in speech patterns [12]. However, there was no evidence to suggest that men differentially targeted women for this speech, nor was there any evidence that women were more likely to target men. In fact, women were found to be less likely to ask men questions than their male audience counterparts (the opposite direction than what was predicted). While this might be due to gendered expectations of deference or perhaps the interactions between gender and academic rank, it could also be due to the combined effects of men being more likely to ask questions in general and trend towards asking more questions to male speakers than to female speakers. In addition to this, a post-hoc analysis revealed that men were found to be more likely to ask questions when they were in rooms with relatively more women.

Due to the structure of AAA sessions, the analytical approach taken in this paper was more complex than originally intended. In AAA sessions, presentations last 15 minutes and all questions are typically held until the final block of the session. Thus, individuals would be simultaneously presented the opportunity to ask numerous male and female presenters a question. The authors attempted to model the likelihood of an audience member asking a question for each possible audience member/speaker dyad, with audience member and speaker as crossed random effects. This created a very large dataset and most of the resulting analyses resulted in convergence problems. This unnecessary complexity could be avoided in future research by focusing on conferences in which it is standard to solicit questions after each presentation.

It is important to note that many “critical” comments may actually be offered out of a spirit of collaboration and helpfulness. Indeed, one can argue that one of the primary purposes of presenting at an academic conference is to seek constructive, critical commentary. Having participated in numerous conference proceedings, however, the authors can attest that while criticisms often result in positive changes to one’s work, they also tend to evoke a negative emotional response in the moment. Despite possible positive intentions and impacts on research, it is likely that higher rates of critical comments, or a more negative reaction to them, would have a negative impact on one’s experience at conferences and erode one’s self-confidence, potentially leading to an imposter syndrome.

Similarly, it is impossible to ascertain perceived status differences, which likely would impact how one experiences the criticism (and represents a defining element of the mansplaining phenomenon). However, it would be surprising if presenters on a particular topic held less expertise than the average question asker.

Finally, it is possible that women and men experience criticism differently. If the perceived threat of criticism is in any way calibrated to one’s own propensity to offer it, then women might experience more of a negative response upon being criticized. Evidence suggests that women perceive related behavior, such as interrupting [36] and anger [37], differently than men. Any such differences would lead to disparate effects of men’s greater assertiveness and criticalness even if men speak to men and women in a similar manner.

While this study suffers from some of the elements that have been critiqued in earlier tabulation studies, its focus on speech content, and particularly on critical content, fills an important void in the literature. We provide further evidence that men exhibit a tendency towards assertive and critical speech compared to women. However, results do not suggest a bias in men’s targets of such speech at a major academic conference, failing to support the widely-discussed phenomenon of mansplaining in this context. It is important to note that this study cannot be generalized to speech in general. As previous research has shown, environmental context and relational characteristics are often essential to understanding the relationship between gender and speech. In this study, we focused on an academic conference because of the importance of such venues in professional development, the augmented impact any biases would result in at such venues, and the discrete nature of speech offered during question rounds. Our understanding of this phenomenon would obviously be enhanced by expanding the range of contexts in the future. Furthermore, future studies could improve upon the current research design by interviewing recipients of critical speech to determine the recipients’ perceptions of the exchange as well as any perception of status difference.

Ultimately, we believe that in the exploration of gender discrimination and biases, quantitative studies are an important complement to more experientially-focused research. Such studies can further elucidate the frequency and impacts of discriminatory patterns, highlighting areas that might be in greater need for advocacy; they can assess the effectiveness of such advocacy and chronical societal changes in positive or negative directions. For such complex and socially important topics, the holistic approach that anthropology is founded upon is essential.

## Supporting information

S1 Table Full results of mixed logistic models testing predicted effects for male audience members. (DOCX)

S2 Table Full results of mixed logistic models testing predicted effects for female audience members. (DOCX)

S3 Table Results of standard logistic models testing for general gender effects for male audience members. (DOCX)

S4 Table Results of standard logistic models testing for general gender effects for female audience members. (DOCX)

S5 Table Sensitivity estimates (odds ratios) for logistic models with β = 0.9. (DOCX)

S1 Data Raw data. (XLSX)
