# Supplementary material for: M-AAA-nsplaining: Gender bias in questions asked at the American Anthropological Association’s Annual Meetings
Source: PLoS One. 2019 Jan 18;14(1):e0207691. doi: 10.1371/journal.pone.0207691 (PMC6338375; doi:10.1371/journal.pone.0207691)
Supplement: S1 Table — (DOCX) [file pone.0207691.s001.docx]

Table S1: Full results of mixed logistic models testing predicted effects for male audience members.

|  | Estimate | S.E. | Signif. |
| --- | --- | --- | --- |
| P2a: Ask more ?s to opposite sex than to same sex^a^ |  |  |  |
| Intercept | -1.7248 | 0.4056 | <.0001 |
| Gender=Woman | -0.1570 | 0.2477 | 0.5269 |
| Audience Size | -0.0342 | 0.0112 | 0.0025 |
| Women Present | 0.3236 | 0.1183 | 0.0067 |
|  |  |  |  |
| P2b: Ask more ?s than opposite sex to opposite sex^b^ |  |  |  |
| Intercept | -1.8237 | 0.3857 | <.0001 |
| Gender=Woman | -0.2452 | 0.2148 | 0.2540 |
| Audience Size | -0.0237 | 0.0092 | 0.0101 |
| Women Present | 0.2579 | 0.0969 | 0.0079 |
|  |  |  |  |
| P3a: More ?s to opposite sex critical than to same sex^c^ |  |  |  |
| Intercept | -0.6819 | 0.4662 | 0.1502 |
| Gender=Woman | 0.3795 | 0.6403 | 0.5636 |
|  |  |  |  |
| P3b: More ?s than opposite sex’s ?s critical to opposite sex^d^ |  |  |  |
| Intercept | -0.8274 | 0.3451 | 0.0194 |
| Gender=Woman | -0.04221 | 0.4521 | 0.9260 |
|  |  |  |  |
| P4a: Ask more critical ?s to opposite sex than to same sex^e^ |  |  |  |
| Intercept | -4.5141 | 0.6100 | <.0001 |
| Gender=Woman | 0.0365 | 0.4103 | 0.9292 |
| Women Present | 0.5304 | 0.1944 | 0.0068 |
|  |  |  |  |
| P4b: Ask more critical ?s than opposite sex to opposite sex^f^ |  |  |  |
| Intercept | -2.9079 | 0.2725 | <.0001 |
| Gender=Woman | -0.2765 | 0.3595 | 0.4421 |
|  |  |  |  |

^a^Unit of analysis=Audience member opportunity. Session and Audience Member ID (nested) included as random effects. n=594. Variance of random effect=1.0982.

^b^Unit of analysis=Audience member. Session included as a random effect. n=747. Variance of random effect=0.0251.

^c^Unit of analysis=Question. Speaker ID included as a random effect. Questions directed to entire panels excluded. n=62. Variance of random effect=1.2161.

^d^Unit of analysis=Question. Speaker ID included as a random effect. n=112. Variance of random effect=0.6950.

^e^Unit of analysis=Audience member opportunity. Session and Audience Member ID (nested) included as a random effect. n=594. Variance of random effect=0.7353.

^f^Unit of analysis=Audience member. Session included as a random effect. n=747. Variance of random effect=0.1206.
