# Supplementary material for: M-AAA-nsplaining: Gender bias in questions asked at the American Anthropological Association’s Annual Meetings
Source: PLoS One. 2019 Jan 18;14(1):e0207691. doi: 10.1371/journal.pone.0207691 (PMC6338375; doi:10.1371/journal.pone.0207691)
Supplement: S2 Table — (DOCX) [file pone.0207691.s002.docx]

Table S2: Full results of mixed logistic models testing predicted effects for female audience members.

|  | Estimate | S.E. | Signif. |
| --- | --- | --- | --- |
| P2a: Ask more ?s to opposite sex than to same sex^a^ |  |  |  |
| Intercept | -1.4816 | 0.2681 | <.0001 |
| Gender=Woman | 0.2589 | 0.2158 | 0.2308 |
| Audience Size | -0.0313 | 0.0092 | 0.0007 |
|  |  |  |  |
| P2b: Ask more ?s than opposite sex to opposite sex^b^ |  |  |  |
| Intercept | -0.7827 | 0.3304 | 0.0232 |
| Gender=Woman | -0.5117 | 0.2366 | 0.0309 |
| Audience Size | -0.0402 | 0.0128 | 0.0017 |
|  |  |  |  |
| P3a: More ?s to opposite sex critical than to same sex^c^ |  |  |  |
| Intercept | -1.2200 | 0.5145 | 0.0217 |
| Gender=Woman | 0.3485 | 0.6453 | 0.5939 |
|  |  |  |  |
| P3b: More ?s than opposite sex’s ?s critical to opposite sex^d^ |  |  |  |
| Intercept | -1.2083 | 0.4405 | 0.0086 |
| Gender=Woman | 0.1731 | 0.5752 | 0.7647 |
|  |  |  |  |
| P4a: Ask more critical ?s to opposite sex than to same sex^e^ |  |  |  |
| Intercept | -3.8423 | 0.3219 | <.0001 |
| Gender=Woman | 0.4975 | 0.3978 | 0.2117 |
|  |  |  |  |
| P4b: Ask more critical ?s than opposite sex to opposite sex^f^ |  |  |  |
| Intercept | -3.2924 | 0.3433 | <.0001 |
| Gender=Woman | -0.5188 | 0.4326 | 0.2309 |
|  |  |  |  |

^a^Unit of analysis=Audience member opportunity. Session and Audience Member ID (nested) included as random effects. n=900. Variance of random effect=1.1899.

^b^Unit of analysis=Audience member. Session included as a random effect. n=747. Variance of random effect=0.4841.

^c^Unit of analysis=Question. Speaker ID included as a random effect. Questions directed to entire panels excluded. n=76. Variance of random effect=1.0982.

^d^Unit of analysis=Question. Speaker ID included as a random effect. n=98. Variance of random effect=2.0966.

^e^Unit of analysis=Audience member opportunity. Session and Audience Member ID (nested) included as a random effect. n=900. Variance of random effect=1.9628.

^f^Unit of analysis=Audience member. Session included as a random effect. n=747. Variance of random effect=0.8309.
