# Supplementary material for: M-AAA-nsplaining: Gender bias in questions asked at the American Anthropological Association’s Annual Meetings
Source: PLoS One. 2019 Jan 18;14(1):e0207691. doi: 10.1371/journal.pone.0207691 (PMC6338375; doi:10.1371/journal.pone.0207691)
Supplement: S3 Table — (DOCX) [file pone.0207691.s003.docx]

Table S3: Results of standard logistic models testing for general gender effects for male audience members.

|  | Estimate | S.E. | Signif. |
| --- | --- | --- | --- |
| P2a: Ask more ?s to opposite sex than to same sex^a^ |  |  |  |
| Intercept | -1.6468 | 0.3687 | <.0001 |
| Gender=Woman | -0.0670 | 0.1193 | 0.5742 |
| Audience Size | -0.0346 | 0.0100 | 0.0006 |
| Women Present | 0.2966 | 0.1104 | 0.0072 |
|  |  |  |  |
| P2b: Ask more ?s than opposite sex to opposite sex^b^ |  |  |  |
| Intercept | -1.9360 | 0.3648 | <.0001 |
| Gender=Woman | -0.1209 | 0.1071 | 0.2590 |
| Audience Size | -0.0236 | 0.0089 | 0.0078 |
| Women Present | 0.2539 | 0.0931 | 0.0064 |
|  |  |  |  |
| P3a: More ?s to opposite sex critical than to same sex^c^ |  |  |  |
| Intercept | -0.5607 | 0.2632 | 0.0332 |
| Gender=Woman | 0.1812 | 0.2632 | 0.4912 |
|  |  |  |  |
| P3b: More ?s than opposite sex’s ?s critical to opposite sex^d^ |  |  |  |
| Intercept | -0.8673 | 0.2085 | <.0001 |
| Gender=Woman | -0.0490 | 0.2085 | 0.8142 |
|  |  |  |  |
| P4a: Ask more critical ?s to opposite sex than to same sex^e^ |  |  |  |
| Intercept | -4.4577 | 0.5893 | <.0001 |
| Gender=Woman | 0.0222 | 0.2033 | 0.9132 |
| Women Present | 0.5266 | 0.1883 | 0.0052 |
|  |  |  |  |
| P4b: Ask more critical ?s than opposite sex to opposite sex^f^ |  |  |  |
| Intercept | -3.0514 | 0.1790 | <.0001 |
| Gender=Woman | -0.1427 | 0.1790 | 0.4252 |
|  |  |  |  |

^a^Unit of analysis=Audience member opportunity. n=594.

^b^Unit of analysis=Audience member. n=747.

^c^Unit of analysis=Question. Questions directed to entire panels excluded. n=62.

^d^Unit of analysis=Question. n=112.

^e^Unit of analysis=Audience member opportunity. n=594.

^f^Unit of analysis=Audience member. n=747.
