# Supplementary material for: M-AAA-nsplaining: Gender bias in questions asked at the American Anthropological Association’s Annual Meetings
Source: PLoS One. 2019 Jan 18;14(1):e0207691. doi: 10.1371/journal.pone.0207691 (PMC6338375; doi:10.1371/journal.pone.0207691)
Supplement: S4 Table — (DOCX) [file pone.0207691.s004.docx]

Table S4: Results of standard logistic models testing for general gender effects for female audience members.

|  | Estimate | S.E. | Signif. |
| --- | --- | --- | --- |
| P2a: Ask more ?s to opposite sex than to same sex^a^ |  |  |  |
| Intercept | -1.2665 | 0.2071 | <.0001 |
| Gender=Woman | 0.1181 | 0.1049 | 0.2603 |
| Audience Size | -0.0309 | 0.0083 | 0.0002 |
|  |  |  |  |
| P2b: Ask more ?s than opposite sex to opposite sex^b^ |  |  |  |
| Intercept | -1.0109 | 0.2302 | <.0001 |
| Gender=Woman | -0.1977 | 0.1138 | 0.0824 |
| Audience Size | -0.0391 | 0.00959 | <.0001 |
|  |  |  |  |
| P3a: More ?s to opposite sex critical than to same sex^c^ |  |  |  |
| Intercept | -0.9784 | 0.2630 | 0.0002 |
| Gender=Woman | 0.0776 | 0.2630 | 0.7678 |
|  |  |  |  |
| P3b: More ?s than opposite sex’s ?s critical to opposite sex^d^ |  |  |  |
| Intercept | -1.0723 | 0.2319 | <.0001 |
| Gender=Woman | 0.0537 | 0.2319 | 0.8168 |
|  |  |  |  |
| P4a: Ask more critical ?s to opposite sex than to same sex^e^ |  |  |  |
| Intercept | -3.4323 | 0.1943 | <.0001 |
| Gender=Woman | 0.2382 | 0.1943 | 0.2203 |
|  |  |  |  |
| P4b: Ask more critical ?s than opposite sex to opposite sex^f^ |  |  |  |
| Intercept | -3.4312 | 0.2121 | <.0001 |
| Gender=Woman | -0.2393 | 0.2121 | 0.2592 |
|  |  |  |  |

^a^Unit of analysis=Audience member opportunity. n=900.

^b^Unit of analysis=Audience member. n=747.

^c^Unit of analysis=Question. Questions directed to entire panels excluded. n=76.

^d^Unit of analysis=Question. n=98.

^e^Unit of analysis=Audience member opportunity. n=900.

^f^Unit of analysis=Audience member. n=747.
