# Supplementary material for: M-AAA-nsplaining: Gender bias in questions asked at the American Anthropological Association’s Annual Meetings
Source: PLoS One. 2019 Jan 18;14(1):e0207691. doi: 10.1371/journal.pone.0207691 (PMC6338375; doi:10.1371/journal.pone.0207691)
Supplement: S5 Table — (DOCX) [file pone.0207691.s005.docx]

Table S5: Sensitivity Estimates (Odds Ratios) for Logistic Models with β=0.9.

|  | Male Audience Member | | Female Audience Member | |
| --- | --- | --- | --- | --- |
|  | α=0.05 | α=0.10 | α=0.05 | α=0.10 |
| P2a: Ask more ?s to opposite sex than to same sex^a^ | 1.993 | 1.871 | 1.794 | 1.701 |
| P2b: Ask more ?s than opposite sex to opposite sex^b^ | 1.930 | 1.816 | 1.889 | 1.783 |
| P3a: More ?s to opposite sex critical than to same sex^c^ | 3.600 | 3.160 | 4.956 | 4.185 |
| P3b: More ?s than opposite sex’s ?s critical to opposite sex^d^ | 3.643 | 3.200 | 4.035 | 3.516 |
| P4a: Ask more critical ?s to opposite sex than to same sex^e^ | 3.020 | 2.745 | 2.510 | 2.318 |
| P4b: Ask more critical ?s than opposite sex to opposite sex^f^ | 3.511 | 3.139 | 2.712 | 2.496 |

^a^For men, n=594; controls include Audience Size and Number of Female Speakers. For women, n=900; controls include Audience Size.

^b^For men, n=747; controls include Audience Size and Number of Female Speakers. For women, n=747; controls include Audience Size.

^c^Questions directed to entire panels excluded. For men, n=62. For women, n=76.

^d^For men, n=112, no controls. For women, n=98, no controls.

^e^For men, n=594; controls include Number of Female Speakers. For women, n=900, no controls.

^f^For men, n=747, no controls. For women, n=747, no controls.
